# The Need to Look for Visual Deficit After Stroke in Children

**DOI:** 10.3389/fneur.2020.00617

**Published:** 2020-07-02

**Authors:** Judith Luckman, Sylvie Chokron, Shalom Michowiz, Eugenia Belenky, Helen Toledano, Alon Zahavi, Nitza Goldenberg-Cohen

**Affiliations:** ^1^Department of Radiology, Rabin Medical Center – Beilinson Hospital, Petach Tikva, Israel; ^2^Responsable de l'Unité Fonctionnelle Vision et Cognition Service de Neurologie Fondation Ophtalmologique Rothschild, Paris, France; ^3^Department of Neurosurgery, Hadassah Hebrew University, Jerusalem, Israel; ^4^Pediatric Oncology, Schneider Children's Medical Center of Israel, Petach Tikva, Israel; ^5^Department of Ophthalmology, Rabin Medical Center – Beilinson Hospital, Petach Tikva, Israel; ^6^Sackler Faculty of Medicine, Tel Aviv University, Tel Aviv, Israel; ^7^Krieger Eye Research Laboratory, Felsenstein Medical Research Center, Rabin Medical Center, Petach Tikva, Israel; ^8^Department of Ophthalmology, Bnai Zion Medical Center, Haifa, Israel; ^9^Faculty of Medicine, Technion – Israel Institute of Technology, Haifa, Israel

**Keywords:** arterial stroke, children, ophthalmological complication, eye exam, cortical visual impairment

## Abstract

**Purpose:** To evaluate the role of the ophthalmologist in the management of children with arterial stroke, at presentation and during follow-up.

**Methods:** This retrospective case series comprised children with arterial stroke who were followed for at least 12 months in a tertiary pediatric medical center in 2005–2016. Demographic data and findings on radiological neuroimaging and ophthalmological and neurological examination were retrieved from the medical files.

**Results:** The cohort included 26 children with stroke. Underlying disorders included metabolic syndrome (*n* = 5, 19.2%), cardiac anomaly or Fontan repair (*n* = 3 each, 11.5%), vascular anomaly (*n* = 3, 11.5%), head trauma with traumatic dissection (*n* = 3, 11.5%), and hypercoagulability (*n* = 1, 3.8%); in eight patients (30.8%), no apparent cause was found. Eleven patients (42.3%) had a non-ophthalmological neurological deficit as a result of the stroke. Eye examination was performed in nine patients (34.6%) during follow-up. Ophthalmological manifestations included hemianopic visual field defect in seven patients (7.7%) and complete blindness and poor visual acuity in one patient each (3.8%). At the last visit, no change in visual function was detected.

**Conclusion:** The variable etiology and presentation of pediatric stroke may mask specific visual signs. Children with arterial stroke should be referred for early ophthalmological evaluation and visual rehabilitation.

## Introduction

Arterial brain stroke is relatively rare in children (age 30 days to 18 years), with international incidence rates ranging from 1.3 to 13 per 100,000 (1–3). It differs from venous stroke in clinical presentation, signs and symptoms, outcome, and consequences. Risk factors are numerous and vary by age; many are unique to children (4–6). The main ones are congenital heart disease, hematologic disorders, and traumatic arterial dissection (7). However, risk factors are missed in about one-third of children with stroke, such that many cases may go undiagnosed or misdiagnosed (1). The diagnosis is confirmed by neuroimaging, either computed tomography or magnetic resonance imaging with venographic sequencing. Treatment requires a combination of medical and surgical approaches in a multidisciplinary pediatric hospital setting. Arterial brain stroke in children can lead to significant morbidity and mortality. Prognosis depends on the extent of vessel and brain parenchymal involvement and the timeliness of diagnosis and therapy.

Visual impairments are very frequent in adults after stroke (8). The most common visual field defect is homonymous hemianopia due to a retrochiasmic lesion, reported in 30% of adults after stroke and in 60% after posterior arterial stroke (9–11). Visual impairments may also be cerebral, affecting the post-chiasmic pathways involved in integrating, and interpreting incoming visual information. Cerebral visual impairment (CVI) may be present even if the cerebral damage itself is not visible by modern imaging modalities. Additionally, CVI may cause very low visual acuity in both eyes, or significant visual difficulties may be evident in the context of normal visual acuity (12). Recent studies in adults with post-stroke CVI found that the lesion was invisible in more than 30% of cases (13).

In children, visual signs may be present in the course of arterial stroke, but the rate of referral for ophthalmological evaluation is unclear. Although systematic protocols to search for visual deficits are lacking, there is no anatomical or functional reason to expect that the high occurrence of stroke-associated ophthalmological or cerebral visual deficits in adults is not also true for children (14). Visual deficits may impact development in children, and when unrecognized, can disturb post-stroke rehabilitation with an adverse effect on behavior, cognition, learning, and social interaction (15). Therefore, it is important that clinicians address visual function in children after arterial brain stroke.

The aim of the present study was to describe the 10-year experience of a tertiary pediatric ophthalmology service with children with arterial stroke, at presentation and during follow-up.

## Materials and Methods

The study was conducted in a tertiary university-affiliated pediatric medical center. A search was conducted of the computerized databases of the hospital and the radiology and ophthalmology services for children aged 2–18 years who were diagnosed with arterial brain stroke between January 2005 and February 2016 and followed for at least 12 months after the acute event. Children with venous stroke or perinatal stroke were excluded. Data on demographics and findings on ophthalmological and neurological examination and neuroimaging were collected from the medical files.

The study protocol was approved by the local Institutional Review Board which waived the need for informed consent.

## Results

**Figure 1:**
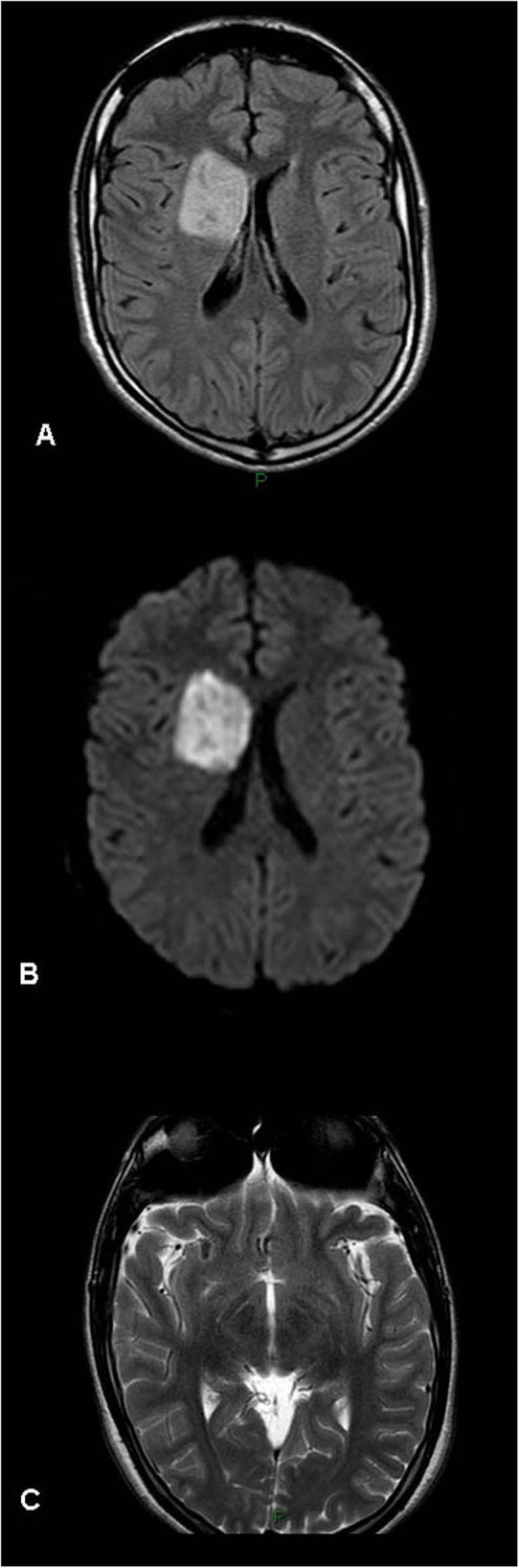
Acute right basal ganglia infarct – magnetic resonance (MR) findings (patient 7, Table 1). **(A)** FLAIR. **(B)** DWI. **(C)** T2 imaging. Right acute basal ganglia infarct with right M2 segment pseudoaneurysm visualized on the T2 weighted imaging, consistent with the patient history of Marfan disease.

**Figure 2:**
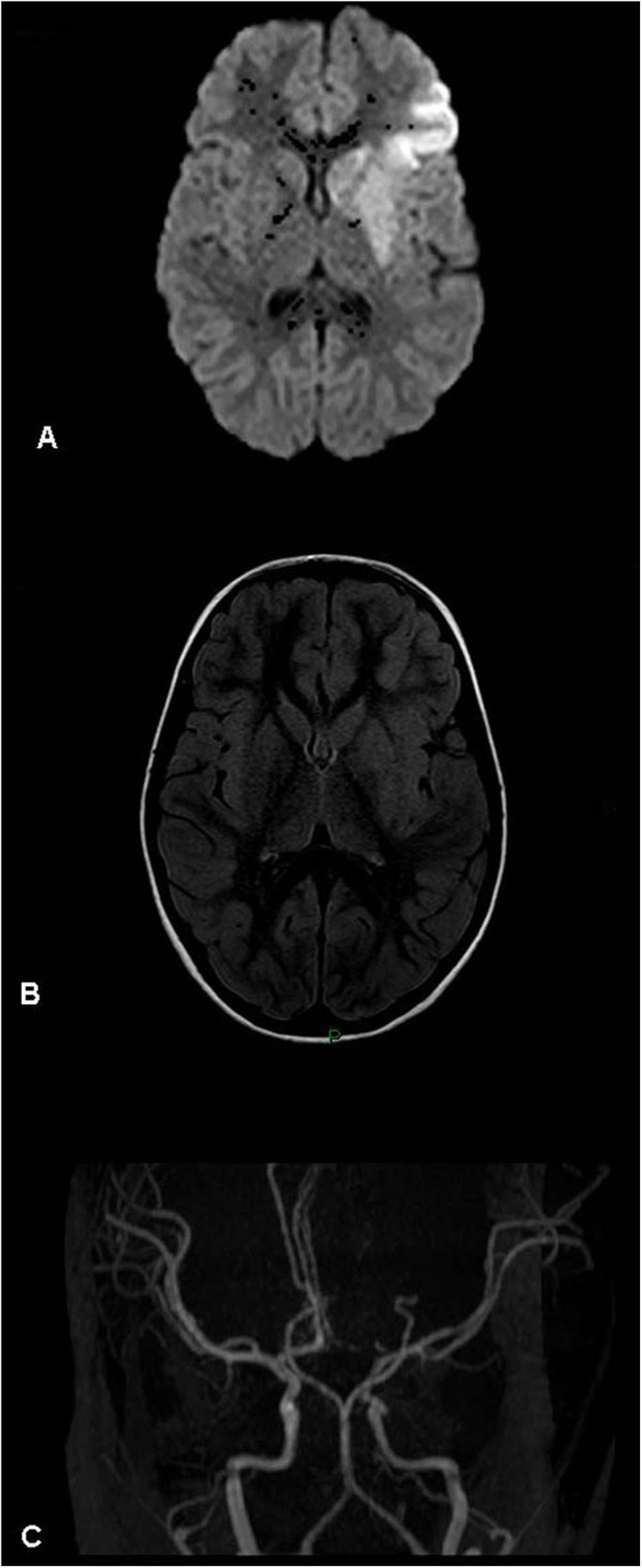
Acute left basal ganglia infarct – magnetic resonance (MR) findings (patient 10, Table 1). **(A)** Diffusion weighted image. **(B)** MR FLAIR. **(C)** MR angiogram (3D-TOF). Left basal ganglia and perisylvian acute stroke visualized mostly on diffusion weighted imaging. No mass effect. The angiogram shows an atretic left A1 segment (anatomic variant). No evidence of arterial occlusion. Left M2 segment focal narrowing is visualized.

**Figure 3:**
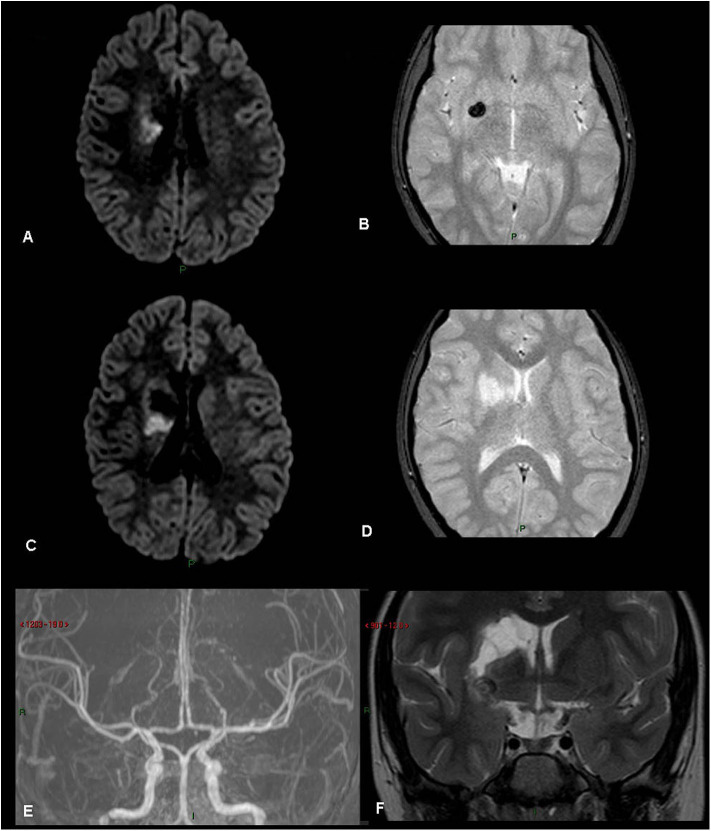
Rt basal ganglia old infarct with hemorrhage. (patient 21, Table 1) **(A)** FLAIR. **(B)** Gradient Echo T2. **(C)** FLAIR. **(D)** Gradient Echo T2. **(E)** MRA TOS. **(F)** Coronal T2. Old right basal ganglia infarct with evidence of hemosiderin, old hemorrhage.

**Figure 4:**
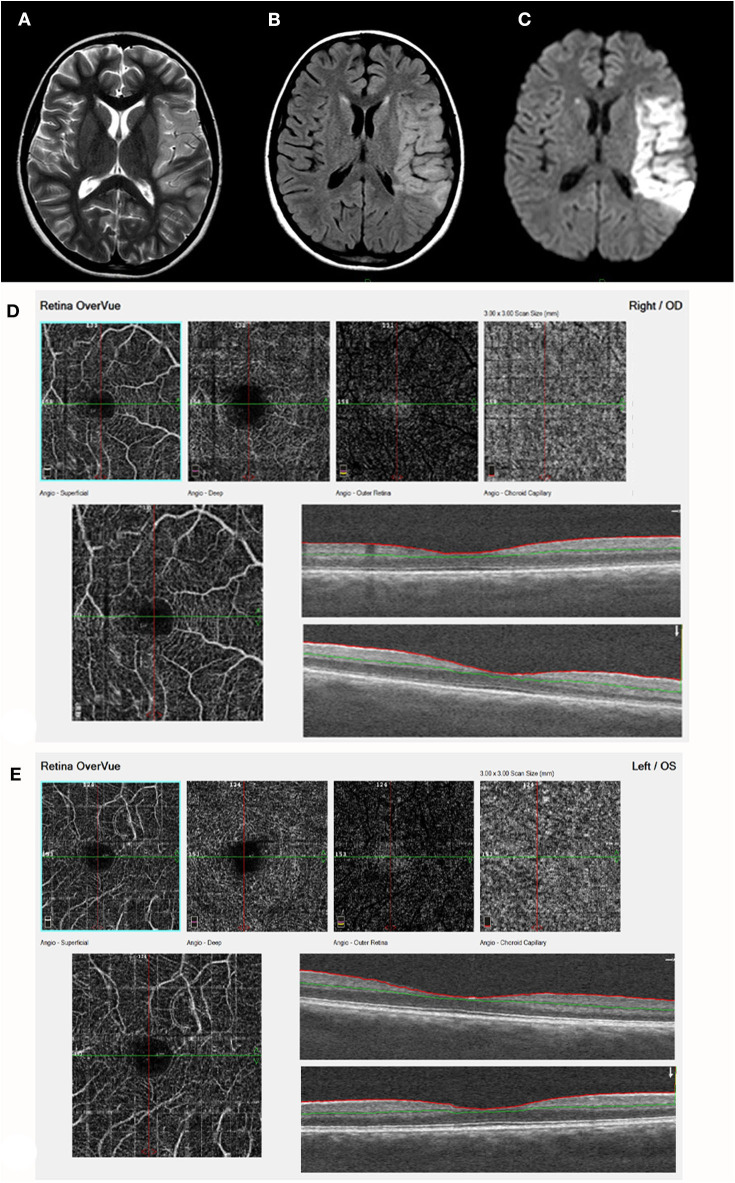
Acute stroke, left MCA territory (patient 17, Table 1). **(A)** T2. **(B)** FLAIR. **(C)** DWI - axial studies, acute frontal stroke with restriction on DWI. A left sylvian MCA acute infarct is visualized with restricted diffusion. The finding is visualized on a T2, FLAIR, and DWI images. **(D)** (right eye) and **(E)** (Left eye) demonstrate by ocular coherence tomography-angiography (OCT-A). Showing Segmentation of different vascular layers using OCTA: left to right: superficial plexus, deep plexus of the inner retina, outer retina (shows absence of vasculature), and choriocapillaris layer. Severe attenuation of the retinal arteries in the superficial layer is demonstrated in both eyes, Lower line: superficial retina and OCT b-scan image of the retina, showing normal retinal layers.

A total of 53 children were diagnosed with arterial brain stroke during the study period (incidence of 4.81 per year). Twenty-seven were excluded from the present study: 22 with perinatal stroke, 3 with venous stroke, and 2 with stroke and pernicious anemia who were missing data. The remaining 26 children formed the study cohort. They included 18 male (69.2%) and 8 female patients of mean age 8.4 years (range 3.2–17.3). Their clinical data are summarized in Table 1, Figures 1–4.

**Table 1:** Data for 26 patients with cerebral arterial stroke.

**Pt. no**.	**Age (yrs)**	**Dx. site**	**Cause**	**VA deficit**	**VF deficit**	**Ophthalmoplegia**	**ON appearance**	**Neurological deficit**
1	6–10	Pons - Lt. vertebralis	Coagulopathy	20/20 20/20	No	No	Normal	Rt. hemiparesis
2	6–10	MCA - Bil. recurrence	Congenital	NA	NA	NA	NA	Rt. Hemiparesis
3	0–5	CVA MCA metabolic	Congenital	NA	NA	NA	NA	NA
4	6–10	Rt. MCA+carotid dissection	Idiopathic	No	No	No	Normal	Lt. Hemiparesis
5	0–5	Rt. MCA	Cardiac	NA	NA	NA	NA	Lt. hemiparesis
6	6–10	Bil. PCA	Trauma	20/20 20/20	Rt. Hemianopia	No	Normal	None
7	16–20	Rt MCA	Congenital	20/20 20/20	No	No	Normal	Lt. facialis (central)
8	6–10	Lt. MCA	Surgery	NA	NA	NA	NA	NA
9	16–20	Lt. ACA	Cardiac	NA	NA	NA	NA	AphasiaHeadache
10	16–20	Lt. MCA	Idiopathic	NA	NA	NA	NA	Lt. hemiparesis
11	6–10	Rt. MCA	Trauma	NA	NA	NA	NA	NA
12	11–15	Lt. PCA	Idiopathic	NA	NA	NA	NA	Rt. hemiparesis
13	6-10	Lt. MCA	Idiopathic	NA	NA	NA	NA	Headache Eye pain Facial asymmetry
14	6–10	Rt. MCA	Idiopathic	20/20 20/20	No	No	Swollen, structural (no papilledema)	Rt. hemiparesis+Lt. facialis (central)
15	6–10	Lt. MCA	Idiopathic	NA	NA	NA	NA	Rt. hemiparesis
16	6–10	Lt. MCA	Congenital	NA	NA	NA	NA	Rt. hemiparesis
17	11–15	Lt. MCA	Surgery for aortic coarctation and aneurysm	20/133 20/33	Rt. Hemianopia	Full motility, reduced Rt. saccades	Normal disc with severe attenuated retinal arteries	Rt. hemiparesis+Rt. facialis (central) Hypertension
18	6–10	Lt. MCA	Idiopathic	NA	NA	NA	NA	Rt. hemiparesis
19	0–5	Lt. MCA	Cardiac	NA	NA	NA	NA	Rt. hemiparesis
20	0–5	Lt. MCA	Congenital	NA	NA	NA	NA	Focal seizure
21	6–10	Rt. MCA	Congenital	NA	NA	NA	NA	Lt. hemiparesis
22	0–5	Rt. MCA	Congenital Alagille syndrome	20/20 20/20	NA	NA	PTC with papilledema, resolved	Nones/p superficial temporal artery to middle artery bypass
23	6–10	Lt. MCA	Congenital	NA	NA	NA	NA	Rt. hemiparesis
24^*^	0–5	Bil. PCA	Surgery for cardiac anomaly	NLP NLP	no fields	No	Normal	No neurological deficit
25	0–5	Lt. MCA	Intracranial surgery chiasmal glioma	20/300 20/300	Rt. Hemianopia	Rt. gaze palsy+exotropia (resolved)	Bil. optic nerve atrophy (temporal)	Rt. hemiparesis+Rt. neglect
26	11–15	Lt. PCA thalamus	Intracranial surgery hypothalamic/ chiasmal glioma (PXA)	CF CF	Rt. Hemianopia	Rt. gaze palsy+ exotropia+Lt. hypertropia	Normal	Rt. Hemiparesis Mutism

*ACA, anterior cerebral artery; Bil, bilateral; Dx, diagnosis; Lt, left; MCA, middle cerebral artery; NA, not applicable [did not have an eye exam]; NLP, no light perception; ON, optic nerve; PCA, posterior cerebral artery; PXA, pleomorphic xanthoastrocytoma; Rt, right; VA, visual acuity; VF, visual field*.

* *The patient had visual deficit but no other neurological deficit*.

Stroke occurred in the anterior circulation territory in 21 patients (80.8%) and in the posterior circulation territory in 5 (19.2%). It was attributed to congenital metabolic syndrome in five patients (19.2%), cardiac anomaly or major repair of a cardiac anomaly in 3 each [23.1%], vascular anomaly in 3 (11.5%), intracranial tumor resection in 2 (7.7%), head trauma with traumatic dissection in 2 (7.7%), and hypercoagulability in 1 (3.8%); in seven patients (26.9%), no apparent cause was found. Twenty patients (76.9%) had a non-visual/non-ophthalmological neurological deficit manifesting as hemiparesis/plegia with or without central VII nerve palsy.

Nine patients (34.6%) underwent ophthalmological evaluation at presentation. Findings included complete cortical blindness secondary to bilateral stroke in 1 (3.8%), unilateral visual field defect (hemianopia) in 4 (15.4%), reduced visual acuity in 3 (11.5%), and gaze paralysis in 2 (7.7%). The patient with bilateral stroke had had multiple uneventful cardiac catheterizations but was rendered completely blind following Fontan repair of cardiac anomalies.

During follow-up, one patient with Marfan syndrome had recurrent transient ischemic attacks, one patient with a hypoplastic left heart had recurrent stroke, and one patient with a hypoplastic left heart underwent arterial bypass, with no recurrences during a 15-year follow-up. An improvement in visual acuity and gaze paralysis was noted in the child in whom the stroke occurred after resection of a suprasellar tumor. There was no change in visual findings during follow-up in any of the other children with documented visual symptoms at presentation.

## Discussion

This study describes the visual findings at presentation and during follow-up in children after arterial brain stroke. Of the total 23 children who experienced stroke during the 10-year study period, 9 (35%) were referred for ophthalmological evaluation at presentation; 5 (56%) had clinically significant findings. The visual loss or visual field defect persisted in all but one patient throughout follow-up.

Most studies of stroke-related visual impairment were performed in adults. Given that the visual pathway accounts for 40% of the brain area, it is not surprising that visual deficits are a frequent sequela of stroke (8–10, 13). In most cases, they occur secondary to damage to the posterior circulation. In children, ischemic strokes usually involve the anterior cerebral circulation (73%) (16). This was true of our cohort as well. Occipital stroke is rare, especially bilaterally. The sole patient in our cohort with bilateral occipital stroke had complete cortical blindness as a result of major pre-stroke Fontan cardiac surgery. According to the recent consensus and evidence-based guidelines (17–19), children with a cardiac source of stroke require different management from children with non-cardiac-related stroke. However, they did not mention visual complications, even though the posterior circulation was sometimes involved.

Visual field defects are more common in post-chiasmal ischemic damage. What appears to be a “unilateral” visual field defect is actually homonymous hemianopia affecting the contralesional visual field involving both eyes. Field defects may be “asymptomatic” or go unnoticed or reported (20), especially when they are not associated with spatial neglect. Children rarely complain that they cannot see things to their right or left, and the variable etiology and presentation may mask specific ophthalmological signs. In some cases, homonymous hemianopia may be detected only during a targeted exam, when it is specifically sought; for example, when the child exhibits truncal turning to examine the whole field of vision or repeatedly bumps into objects on the same side. It is noteworthy that even when visual acuity is normal, children can have impaired vision due to lesions from the chiasm toward the primary visual cortices (21, 22). CVI arises as a consequence of damage or disorder of the brain. It may cause very low visual acuity in both eyes, or alternatively, significant visual difficulties may be evident in the context of normal visual acuity (12). This is particularly important in young children who cannot verbalize or fail to recognize visual impairments.

Kieslich et al. (23) suggested that a detailed medical history of the days before stroke manifestation may identify more traumatic events, especially in idiopathic stroke. In our study, 2 children had a history of trauma prior to the development of an arterial stroke. One of them presented to the emergency room with anisocoria and was diagnosed with Horner syndrome due to carotid dissection. On follow-up, when better co-operation with the examiner achieved, traumatic optic neuropathy associated with monocular visual loss was diagnosed. The other patient had minor trauma and was also diagnosed with carotid dissection, but without visual complications.

In another study, of 90 children who experienced stroke at age 3 months to 15 years (median 5 years) and were followed for a median time of 3 years, only 13 (14%) had no residual impairments (16). Poor outcome with impairment in daily life activity was reported in 53 (60%) children. Surprisingly, however, no attention was addressed to visual outcome, and parents and therapists were not asked about vision impairment.

Our extensive search of the literature did not reveal data on diagnostic, follow-up, or treatment protocols for children with stroke in terms of visual pathologies. Further studies are urgently needed to improve our understanding of vision impairments in children with arterial brain stroke, particularly CVI. This will lead to better and earlier diagnosis and treatment, with optimal differentiation of visual impairments from stroke-induced neurological impairments (21, 22). Furthermore, systematic visual evaluation of children after arterial stroke will prevent the secondary developmental deficits due to CVI.

The main limitation of the present study is the retrospective design which harbors several potential biases. In addition, the sample size was small. However, given that the study was conducted over a 10-year period in a major referral medical center, we assume the low number reflects the real incidence of pediatric arterial brain stroke. Only one child was excluded due to missing data, and none was lost to follow-up.

In conclusion, the variable etiology and presentation of arterial stroke in children may mask specific ophthalmological and visual impairments. At present, visual function is hardly examined, and then usually only after disease stabilization. Eye and visual examination, including fundus examination, visual fields, visual acuity, and visual cognition, is important for proper rehabilitation and should be systematized as an essential part of the initial evaluation and follow-up of all children with an arterial ischemic event in the brain.

## Data Availability Statement

All datasets generated for this study are included in the article/supplementary material.

## Ethics Statement

The studies involving human participants were reviewed and approved by Schneider Children's Medical Center of Israel Institutional Review Board Ethics Committee. Written informed consent from the participants' legal guardian/next of kin was not required to participate in this study in accordance with the national legislation and the institutional requirements.

## Author Contributions

NG-C, EB, and JL: design of the study. NG-C, EB, and AZ: collection of data. NG-C, SC, HT, EB, JL, AZ, and SM: analysis and interpretation. NG-C, SC, EB, AZ, and SM: preparation of the manuscript. All authors: review and approval of the manuscript.

## Conflict of Interest

The authors declare that the research was conducted in the absence of any commercial or financial relationships that could be construed as a potential conflict of interest.
